# Pathological features of early pregnancy disorders in women living at high altitude in KSA

**DOI:** 10.1016/j.jtumed.2022.10.010

**Published:** 2022-11-22

**Authors:** Khalid Nafie, Abdulkarim Hasan, Wesam K. Alshakhrit, Amal Ismail, Osama Abbadi

**Affiliations:** a Pathology and Laboratory Department, Prince Mishari bin Saud Hospital, Ministry of Health, Baljurashi, KSA; b Pathology Department, Faculty of Medicine, Al-Azhar University, Cairo, Egypt; c Obstetrics and Gynecology Department, Prince Mishari bin Saud Hospital, Ministry of Health, Baljurashi, KSA; d Pharmacy Practice- Pathophysiology Department, Unaizah College of Pharmacy, Qassim University, KSA; e Biochemistry Department, Faculty of Medicine, Omdurman Islamic University, Sudan

**Keywords:** إجهاض, نقص الأكسجة, الارتفاع عن سطح البحر, علم الأمراض, Abortion, High-altitude, Hypoxia, Miscarriage, Products of conception

## Abstract

**Objectives:**

Pregnancy at high altitudes is relatively challenging because of hypobaric hypoxia, which is associated with a smaller uterine artery diameter and diminished blood flow. Here, we investigated the histopathological characteristics of early pregnancy disorders among pregnant women living in a high-altitude region (approximately 2200 m above sea level).

**Methods:**

This cross-sectional study used retrospective data collection from a single tertiary hospital in a high-altitude region in KSA. Age and histopathology were analyzed in 495 women diagnosed with early pregnancy disorders (mainly miscarriage) in 2018–2020.

**Results:**

Approximately one-fifth of pregnancies in this high-altitude region were lost before 24 weeks’ gestation, whereas 1150/6044 experienced miscarriage; 495 samples from those participants were sent for histopathological analysis. A total of 269 (54.34%) patients were younger than 35 years. Missed miscarriages accounted for 49.3% of miscarriages, followed by inevitable miscarriages (34.2%), which had a relatively higher frequency among mothers older than 35 years. The correlation between age and inevitable miscarriage was significant; ectopic pregnancy was diagnosed in 6.8%, molar pregnancy was detected in 6.26%, and blighted ovum was observed in 3.4%.

**Conclusion:**

The miscarriage rate in this high-altitude region was 19% of all pregnancies. Approximately half of the affected women were in their 30s. Missed miscarriage cases were relatively high in this region. The percentage of molar pregnancy was higher than those reported in prior studies, thus suggesting a need for monitoring and genetic workup practices. Furthermore, studies involving a larger population at high altitudes will be crucial for further risk assessment in addition to national studies on women living at sea level.

## الملخص

**أهداف البحث:**

يعتقد بأن عملية الحمل تمثل إحدى التحديات التي تواجهها النساء اللاتي يقطن الأماكن شديدة الارتفاع عن سطح البحر، وذلك نظرا لصعوبة التأقلم مع معضلة نقص الأكسجين. من خلال هذا البحث تم دراسة مجموعة من الخصائص المرضية النسيجية (الباثولوجية) لبعض اضطرابات الحمل التي تتضمن فقدان الحمل (الإجهاض) بين النساء الحوامل اللائي يقطن منطقة مرتفعة عن سطح البحر بحوالي 2200 متر بالمملكة العربية السعودية.

**طرق البحث:**

دراسة مقطعية تم خلالها جمع البيانات بأثر رجعي من مستشفى واحد واقع في منطقة مرتفعة وذلك خلال عامي 2018-2020 .

**النتائج:**

أسفرت النتائج عن أن نسبة تقدر بحوالي 55% من النساء اللاتي حدث عندهن حمل غير مكتمل (إجهاض أو حمل العنقودي أو حمل خارج الرحم) كانت أعارهن تقل عن 35 عامًا. وقد دلت الإحصائية على أن نسبة الحمل خارج الرحم وصلت إلى 6.8% من تلك الاضطرابات. وقد بلغ معدل الإجهاض 19% من إجمالي عدد حالات الحمل التي تمت دراستها

**الاستنتاجات:**

توصي الدراسة الباحثين باستكمال وتواصل الدراسات في هذا الشأن وأن تشتمل دراساتهم المستقبلية على عدد أكبر من السيدات الحوامل في تلك المناطق شديدة الارتفاع عن سطح الأرض مستعينين بالتحليلات الجينية وغيرها من الوسائل التي تساعد على التوصل إلى نتائج أكثر دقة والتعرف على الأسباب وطرق الوقاية من تلك الاضطرابات، إضافة إلى الحاجة إلى المزيد من الدراسات المماثلة بمناطق تقع على مستوى سطح البحر.

## Introduction

Miscarriage within the first 24 weeks of pregnancy might be underestimated because it can occur outside hospitals and clinics and thus might be insufficiently investigated. Aneuploidy is the most common etiology of early miscarriage: 50–70% of miscarriages show a chromosomal or genetic anomaly. Other causes include infection, maternal medical conditions, and uterine structural problems. However, at least 30% of cases are idiopathic.^1^^,^^2^

Pregnancy at high altitudes is considered a challenge. At high altitudes, oxygen availability during early placentation may significantly contribute to miscarriage; high altitude is also associated with a smaller uterine artery diameter and decreased blood flow.^1^^,^^3^

At altitude, the dilatation effect by acetylcholine and bradykinin decreases, owing to the release of nitric oxide synthase inhibitor, thus suggesting predominantly nitric oxide-dependent vasodilation at sea level, where bradykinin vasodilation is not affected by nitric oxide synthase inhibitor, thus indicating impaired nitric oxide signaling at high altitude.^4^

Most high-altitude studies have been conducted among Himalayan people in Tibet, Sherpas, Andeans, and Ethiopian highlanders. These populations show several genetic variants or single nucleotide polymorphisms; consequently, genetic background has been assumed to be the critical factor modifying pregnancy adaptation at high altitudes.^3^

Hypoxia-inducible factor (HIF) is the main pathway through which hypoxia affects more than 100 genes, including vascular endothelial growth factor (*VEGF*), platelet-derived growth factor (*PDGF*), Fms related receptor tyrosine kinase 1 (*FLT*), erythropoietin (*EPO*), cell death-like transforming growth factor alpha (*TGFA*), tumor necrosis factor (*TNF*), matrix metalloproteases (*MMP*), inflammatory cytokines (e.g., *IL6*), vasodilation or constriction like endothelin (*EDN)*, nitric oxide synthase (*NOS*), and protein kinase AMP-activated catalytic subunit alpha 1 (*PRKAA1*).^5^^,^^6^ The population at high altitudes shows different adaptive responses to hypoxia.

This study was aimed at characterizing early pregnancy at high altitude (2200 m above sea level) in KSA and comparing the results with other areas worldwide. Statistically significant differences were revealed, and possible effects and explanations are discussed.

## Materials and Methods

A cross-sectional study using retrospective data collection was conducted at a tertiary care hospital (Prince Mishari bin Saud hospital in Baljurashi) situated in a high-altitude area in the Al-Baha region of KSA. This city is considered a highland plateau, with an altitude 2000–2400 m above sea level. The topography of this area mainly comprises mountains and undulated hills, with strips of fertile valleys.

All medical records for labor and miscarriages in the study period at both obstetrics and pathology departments were verified after local bioethical research committee approval. A total of 495 miscarriages and ectopic pregnancy cases over the course of 2 years were retrieved and categorized according to maternal age, parity, gestational age, pregnancy type, type of miscarriage, and related pathological changes (if found).

For accuracy, histopathological sections (formalin fixed paraffin embedded blocks sectioned at 5 μm, stained with hematoxylin and eosin, mounted with mounting medium, and covered with a cover glass) were reviewed by two experienced pathologists, both of whom were blinded to the patients’ clinical information. Disagreements were resolved through discussion.

The microscopic criteria for hydropic changes were enlarged chorionic villi with edematous stroma, prominent cytotrophoblast, and reduced villous capillaries. Hydatidiform mole showed enlarged edematous villi with variable amounts of trophoblast proliferation/hyperplasia, trophoblast atypia, and intervillous inclusions. In complete mole, all villi were enlarged, with prominent cisternae and circumferential trophoblast proliferation. In partial mole, the villi were irregularly shaped and enlarged with scalloped contours and diminished focal proliferation, admixed with smaller normal-appearing fibrotic villi. A finding of pink fibrinoid material surrounding and widely separating chorionic villi, presenting diffusely, supports the diagnosis of diffuse or massive perivillous fibrin deposition, according to the WHO pathology guidelines.

The inclusion criteria were patients of all ages living in this high-altitude area who experienced miscarriage or ectopic pregnancy during the study period (January 2018–January 2020). The exclusion criteria were patients who experienced pregnancy disorders in the hospital but lived (during the pregnancy) in high altitude areas outside our geographic area (e.g., visitors and referred patients from hospitals in high altitude areas), in addition to patients with no confirmation of their samples by histopathology (e.g., expelled miscarriage without pathological examination). The miscarriage rate was calculated according to pregnancy and miscarriage records in the OB/gyn department for patients meeting the study criteria.

The data were processed in Microsoft Excel (Excel, Microsoft Corporation, Redmond, WA, USA) and transferred to SPSS version 28 (IBM Corporation, Armonk NY, USA). For statistical analysis, frequency and cross-tabulation were run. Tests of significance were performed with the chi-square test, and results were considered significant at a 95% confidence interval. Correlations between numerical data (gestational age at time of abortion, maternal age, and number of pregnancies) were performed with Pearson's correlation coefficient.

## Results

Miscarriage occurred in 1150/6044 pregnant women (19% of all pregnancies during the study period). A total of 495 early pregnancy disorder cases among 6044 pregnancies were received in the histopathology department at our hospital. We retrieved and categorized those cases. The average maternal age at miscarriage was 33.8 years, the average parity was 4.46 (1–11), and the average gestation age was 11.8 weeks. A slightly higher incidence of early pregnancy disorders was observed among mothers younger than 35 years (54.34%), and nearly half of the cases were in mothers 30–39 years old (43%), whereas a small proportion of affected mothers were in their 40s (Table 1).

**Table 1:** Relationships between maternal age and the total number and percentage of abortions, the average gestational age (GA), parity, and types of abortion.

Variable/age grouping		<35 years	>35 years	Total	P values
Number of patients		269	226	495	a**0.0532**
Mean age		28.4	40.2	33.83 (Mean)	0.481
Mean parity		2.9	6.3	4.5	0.804
Mean GA of parity		11.7	11.9	11.8	0.671
Abortion %		54.34	45.66	100	0.385
Type of abortion	Missed %	47.02	51.65	49.34	0.387
	Incomplete %	24.83	30.86	27.85	0.518
	Complete %	6.62	6.04	6.33	0.755
	Total inevitable %	31.45	36.9	34.18	b**0.0413**
	Ectopic %	10.76	2.84	6.8	0.795
	Molar %	7.13	5.4	6.26	0.759
	Blighted ovum %	3.64	3.21	3.42	0.817

a The total P-value is nearly significant, whereas the percentages (last column) are far from the significance threshold.

b Although total inevitable abortion comprises complete and incomplete abortion subtypes, total inevitable abortion has a significant P-value, whereas the complete and incomplete abortion rates have an insignificant P value between age groups.

**Figure 1:**
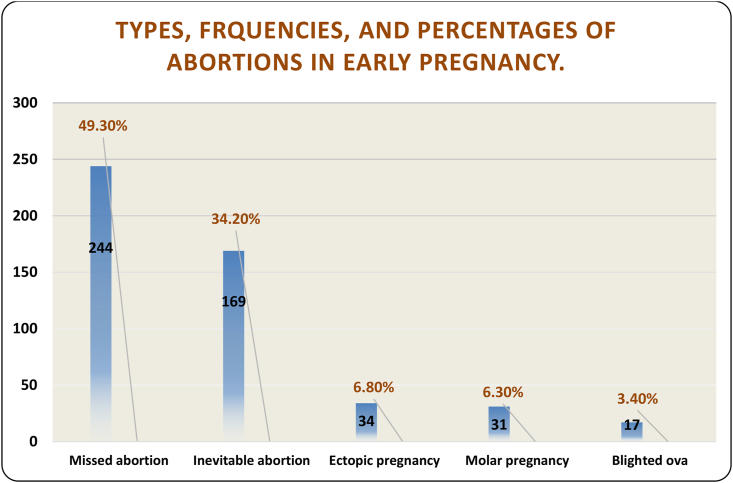
Bar chart showing the frequencies (black) and percentages (red) of each type of miscarriage among the total number of studied cases of abortion.

Most miscarriages were missed miscarriages (49.3%) (Figure 1), followed by inevitable miscarriages (34.2%), including incomplete (27.9%) and complete (6.3%) miscarriages; only a slightly (but statistically significantly) higher fraction of inevitable miscarriages was observed among mothers older than 35 years. The total percentage of ectopic pregnancies (among miscarriages) was 6.8%, 79% of which were among younger mothers (<35 years). However, the incidence was 63 per 1000 deliveries.

Molar pregnancy was observed in 6.3% of the cases, 57% of which were in younger women. The incidence of molar pregnancy was 0.4% (4 per 1000) (Table 1).

A very weak positive correlation (+0.03) was observed between the age of the mothers and the fetal gestational age, whereas a very weak negative correlation (−0.03) was found between the number of pregnancies of each mother and the gestational age at the time of the abortion.

**Figure 2:**
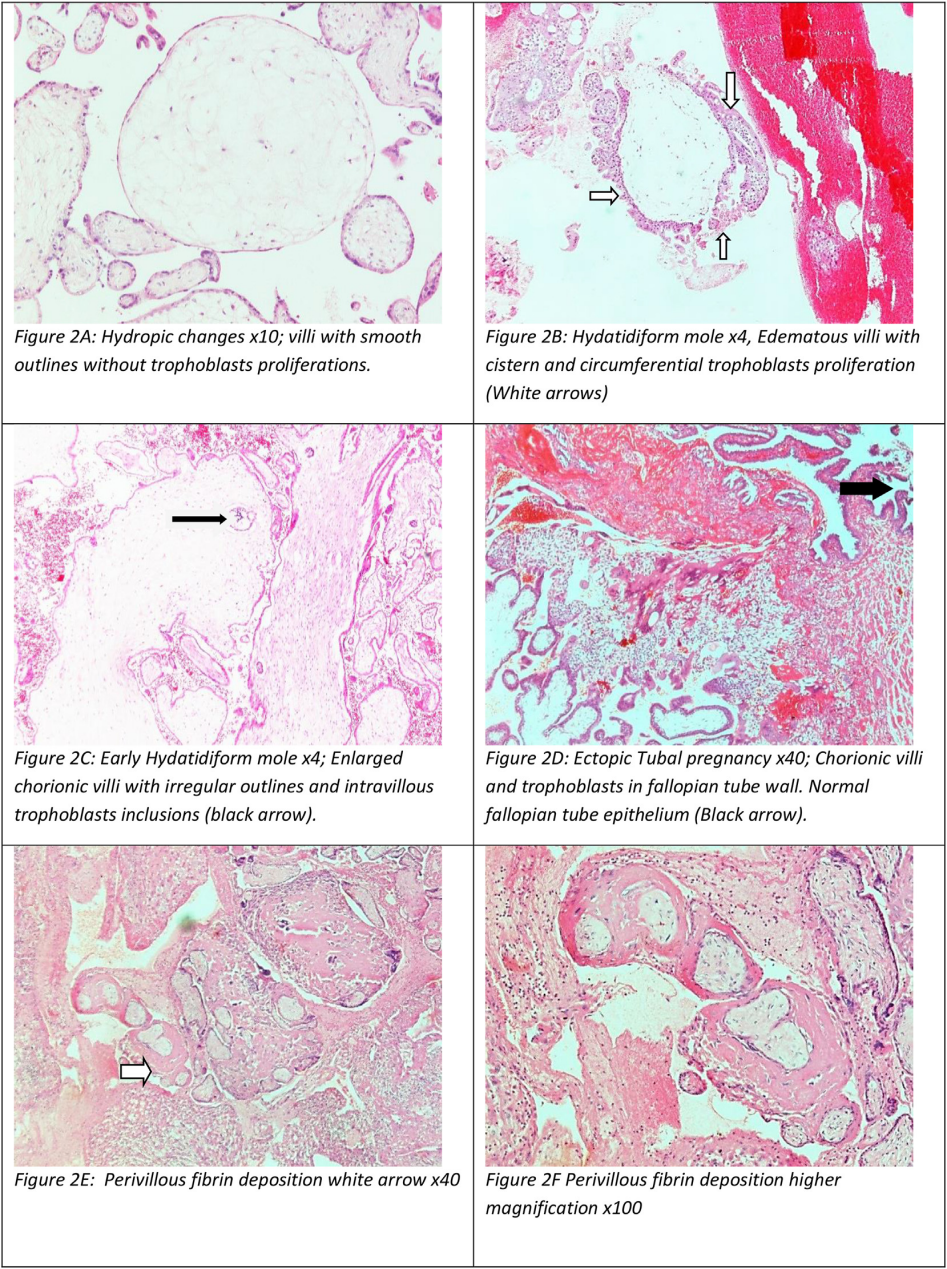
Hematoxylin and eosin staining, depicting different abortus morphological changes seen microscopically.

Regarding histological changes associated with miscarriages, almost half the cases did not show any significant histological changes. In contrast, the remaining cases showed focal hydropic changes (35.2%) (Figure 2), diffuse hydropic changes not associated with molar pregnancy (7.5%), molar pregnancy (7.6%), blighted ovum changes (4.2%), diffuse perivillous fibrin deposition (3.3%), or chorioamnionitis (1.5%). Slightly more than two-thirds of the total hydropic changes (63.8%) and diffuse hydropic changes (68.3%) were seen in missed miscarriage cases. In addition, blighted ovum and chorioamnionitis appeared more prevalent in the same category, whereas diffuse perivillous changes were slightly higher in women with inevitable miscarriage (Table 2). The mean difference in gestational age at the time of abortion was 11.6 for diffuse hydropic change, 10.9 for focal hydropic change, and 12.7 weeks for non-hydropic change; the differences were statistically significant (P = 0.037).

**Table 2:** Characterization and percentages of histological changes associated with abortion type and maternal age.

Histopathological finding/age grouping		Diffuse hydropic %	Focal hydropic %	Total hydropic %	Blighted ovum %	Molar %	Diffuse peri-villous fibrin %	Chorio-amnionitis %	Total (%)
**Distribution in age groups**	<35 years	8.0	31.9	40.0	4.8	9.5	4.6	1.2	100
	>35 years	7.4	35.8	43.0	3.6	6.1	2.3	1.8	100
	Total (both age groups)	7.5	35.2	40.7	4.2	7.6	3.3	1.5	100
	P value	0.735	0.466	0.429	0.776	0.751	0.1	0.907	–
**Distribution according to missed/inevitable cases**	Missed	68.4	62.3	63.8	65	–	42.9	52.9	59.2
	Inevitable	31.6	36.7	36.2	35	–	57.1	47.1	40.8
	P value	**0.002**							
**Distribution in age groups for each histological type**	<35	50	44.9	45.9	55	58.3	62.5	42.8	51.3
	>35	50	55.1	54.1	45	41.7	37.5	57.2	48.7
	P-value	**0.0385**							

Although the difference in the prevalence of histological types in the two age groups was significant (P = 0.0385), the distribution of each histological type within the group was not significant (Table 2). Women older than 35 years had higher percentages of hydropic changes (41.6%) and chorioamnionitis (1.7%), whereas those under 35 years of age had slightly higher percentages of molar pregnancy and diffuse perivillous fibrin deposition.

## Discussion

In this study, miscarriage occurred in 19% of the pregnant women living in this high altitude region during the study period. In Western countries including the United States and the United Kingdom, miscarriage has been found to occur in 11%–22% of clinically recognized pregnancies.^7^ However, Tibetan women in a high-altitude area show a markedly different percentage.^8^

Maternal exposure to a persistent hypoxic environment may lead to injury affecting several vital organs, including failure of normal placental function, which may severely affect the developing fetus, thus leading to intrauterine growth restriction, asphyxia, premature delivery, or perinatal demise.^9^^,^^10^ The main challenges at high altitudes are not only hypoxia, but also high levels of ultraviolet radiation and low temperatures.^10^

Most miscarriages occurred in women in their 30s (43%), in contrast to findings in other studies indicating higher incidence in women around their 40s;^4^^,^^5^^,^^11^^,^^12^ this difference may be attributable to younger ages at marriage in our study. In contrast, a US study has reported that miscarriages occur predominantly among women in their 20s;^13^ however, the study included both types of miscarriage (spontaneous and induced).

We reported an average parity of 4.46 and an 11.8-week average gestational age. Nybo Andersen et al. have observed an increase of the risk of miscarriage in both parous and nulliparous women, regardless of parity, calendar period, or having several previous spontaneous miscarriages.^14^

Among the types of miscarriages, missed miscarriages cases (49.3%) have been found at a significantly higher frequency in some studies, whereas others have found that inevitable miscarriages predominate; however, the results are consistent with the current trend of an increase in missed miscarriages.^13^^,^^15^ In the current study, all ectopic pregnancies were tubal pregnancies, and the incidence was 6.3 per 1000 deliveries. This finding is consistent with those in a similar previous high-altitude area study in Abha, KSA, in 2013, which revealed an incidence of ectopic pregnancy of 7.4 per 1000 live births;^16^ and with a Saudi study in a coastal city in 2011, which revealed 5.8 ectopic pregnancies per 1000 births.^17^ However, we reported a lower incidence in high-altitude areas than a previous study conducted by Zain et al., in 2019, in which the incidence of ectopic pregnancy was 1.2%, a rate similar to that in developing countries (1%–2%).^18^ Al-Turki's study has found a growing trend in the status of ectopic pregnancy in Eastern KSA.^19^

Hydropic changes (edematous chorionic villi) may reflect morphological changes associated with fetal death, hydropic abortions, chorionic villi immaturity, and a vascularization of the villi and, of course, molar pregnancy.^20^^,^^21^ Molar pregnancy must be differentiated from the other causes, because its management and prognosis differ substantially. Features supporting hydropic abortus include uniformly sized chorionic villi, and predominantly round or oval contours, with gradual changes in diameter and no prominent cistern formation or villous trophoblast proliferation/hyperplasia.^22^ Using other techniques, such as immunohistochemistry (e.g., for p57kip2,^23^ which is lost in the trophoblasts of complete molar pregnancy) and/or ploidy analysis, is important in challenging cases and certain entities, such as placental mesenchymal dysplasia.^24^ In our study, hydropic changes appeared more frequently at younger gestational ages (average 10.5 weeks).

Hydatidiform mole arises in the placenta from gestational tissue and can invade the uterus and spread. It is diagnosed through histology, classified into complete and partial, and confirmed by karyotyping.^25^ Molar pregnancy is uncommon in our region.^26^ The rate of molar pregnancy in this study was 6.26% of the total miscarriage cases, with an incidence of 0.4%. A total of 57% of cases were observed in younger women, in agreement with findings from previous studies reporting that extremes of maternal ages (below 20 years or above 40 years) are associated with greater risk of molar pregnancy.^26^^,^^27^ Nonetheless, we observed a higher incidence rate than that of another study in our country at sea level area, which has reported a rate of 0.09%. Although the incidence of gestational trophoblastic diseases has declined with the rapid socio-medical development of the KSA, this study indicated a higher percentage of molar pregnancies than those observed in other studies in high-altitude areas.

In our findings, blighted ovum represented 3.4% of the total miscarriage cases, although obesity and advanced maternal age are well-established factors associated with an embryonic early pregnancy loss. The literature on histological changes associated with miscarriages is very limited. Herein, we investigated the previously described histological features in addition to perivillous fibrin. Most placentas contain some perivillous fibrin deposition, and deposition increases with gestational age; however, massive perivillous fibrin deposition is rare (occurring in less than 1% of deliveries) and should be diagnosed only when perivillous fibrin material involves 25% or more of the chorionic villi,.^28^

To our knowledge, this is the first documentation of pathological evaluation of products of the conception of miscarriage in women in a high-altitude area in KSA revealing histopathological findings, in addition to clinical presentation and ratios of pregnancy disorders. Further studies are needed to identify additional correlations.

This study has several limitations, such as the small sample size involving single-center tertiary hospitals and not involving all hospitals in the high-altitude southern region. In addition, comparing data from KSA was difficult, owing to the lack of karyotyping of the recorded molar pregnancy cases and the lack of study of co-morbidities associated with miscarriage in this high-altitude area. Further large-scale studies are recommended.

## Conclusion

Maternal exposure to a persistent hypoxic environment is thought to affect normal perfusion and may lead to embryological development failure. We concluded that the miscarriage rate in this high-altitude region was 19% of all pregnancies. Approximately half affected mothers were in their 30s. Missed miscarriage cases were seen relatively high in this region. Molar pregnancy was observed at a higher percentage than reported in other studies, thus raising the need for more monitoring and genetic workup practices. Furthermore, studies involving larger populations at high altitudes, in addition to further national studies on women living at high altitudes, will be crucial for further risk assessment.

## Source of funding

This research did not receive any specific grant from funding agencies in the public, commercial, or not-for-profit sectors.

## Conflict of interest

The authors have no conflict of interest to declare.

## Ethical approval

Local bioethical approval was provided from The Prince Mishari bin Saud hospital (number: PMS2202, in 1/2021).

## Authors’ contributions

KN and AH (first authors): Conceived and designed the study, conducted research, provided research materials, collected and organized data and literature review, and participated in data analysis and the writing of all drafts. WA: Participated in study design, collected and organized data, and participated in the literature review. OA: Wrote initial drafts, analyzed statistical study results, and interpreted data. AI: Participated in literature review and discussion of the initial and final drafts. All authors have critically reviewed and approved the final draft and are responsible for the content and similarity index of the manuscript.

## References

[1] Soler A. (2017). Overview of chromosome abnormalities in first trimester miscarriages: a series of 1,011 consecutive chorionic villi sample karyotypes. Cytogenet Genome Res.

[2] Nigro G. (2011). Role of the infections in recurrent spontaneous abortion. J Matern Fetal Neonatal Med.

[3] Grant I. (2020). Parental ancestry and risk of early pregnancy loss at high altitude. Faseb J.

[4] Lorca R.A. (2019). High altitude reduces NO-dependent myometrial artery vasodilator response during pregnancy. Hypertension.

[5] Bigham A.W. (2009). Identifying positive selection candidate loci for high-altitude adaptation in Andean populations. Hum Genom.

[6] Simonson T.S., Malhotra A. (2020). Variability in hypoxic response: could genetics play a role?. J Physiol.

[7] Gaskins A.J., Rich-Edwards J.W., Colaci D.S., Afeiche M.C., Toth T.L., Gillmanet M.W. (2014). Prepregnancy and early adulthood body mass index and adult weight change in relation to fetal loss. Obstet Gynecol.

[8] Dang S.N., Yan H., Zeng L.X. (2006). Epidemiological features of spontaneous abortion among reproductive Tibetan women living at high altitudes areas. Zhonghua Liuxingbingxue Zazhi.

[9] Hutter D., Kingdom J., Jaeggi E. (2010). Causes and mechanisms of intrauterine hypoxia and its impact on the fetal cardiovascular system: a review. Int J Pediatr.

[10] Grant I.D., Giussani D.A., Aiken C.E. (2022). Fetal growth and spontaneous preterm birth in high-altitude pregnancy: a systematic review, meta-analysis, and meta-regression. Int J Gynaecol Obstet.

[11] Shiina Y. (1984). The age incidence of spontaneous abortion. Hokkaido Igaku Zasshi.

[12] Jatlaoui T.C. (2019). Abortion surveillance - United States, 2016. MMWR Surveill Summ.

[13] Othman M. (2018). Rate of different types of abortion in madinah maternity and children hospital, madinah, Saudi Arabia. Investigat Gynecol Res Women Health.

[14] Nybo A.A. (2000). Is maternal age an independent risk factor for fetal loss?. West J Med.

[15] Linnakaari R. (2019). Trends in the incidence, rate and treatment of miscarriage-nationwide register-study in Finland, 1998-2016. Hum Reprod.

[16] Archibong E.I., Sobande A.A. (2000). Ectopic pregnancy in Abha, Saudi Arabia. A continuing conundrum. Saudi Med J.

[17] Aziz S., Al Wafi B., Al Swadi H. (2011). Frequency of ectopic pregnancy in a medical centre, Kingdom of Saudi Arabia. J Pakistan Med Assoc.

[18] Zain H A.R., Mohamed Y Abdallah Y. (2019). Clinical analysis of ectopic pregnancies in Majmaah, Saudi Arabia. Biomed Res.

[19] Abdulaziz Al-Turki H. (2013). Trends in ectopic pregnancies in eastern Saudi Arabia. ISRN Obstet Gynecol.

[20] Benirschke K. (2012). Pathology of human placenta, chapter 14 Histopathological Approach to Villous Alterations.

[21] Naeye R.L. (1983). The clinical significance of placental villous edema. Pediatrics.

[22] Crum Christopher P. (2018). Diagnostic gynecologic and obstetric pathology, chapter 19 placental development and complications of previable pregnancy.

[23] Romaguera R.L. (2004). Molar gestations and hydropic abortions differentiated by p57 immunostaining. Fetal Pediatr Pathol.

[24] Osterheld M.C. (2008). Combination of immunohistochemistry and ploidy analysis to assist histopathological diagnosis of molar diseases. Clin Med Pathol.

[25] Soper J.T. (2021). Gestational trophoblastic disease: current evaluation and management. Obstet Gynecol.

[26] Al-Talib A.A. (2016). Clinical presentation and treatment outcome of molar pregnancy: ten years experience at a Tertiary Care Hospital in Dammam, Saudi Arabia. J Family Community Med.

[27] Kayastha S., Shah L., Mainali S. (2021). Histological examination of tissue obtained in early pregnancy loss. Kathmandu Univ Med J.

[28] Lampi K. (2022). Massive perivillous fibrin deposition of the placenta and pregnancy outcome: a retrospective observational study. Placenta.

